# Ten Years in the Profession

**Published:** 1866-11

**Authors:** J. F. Siddall


### TEN YEARS IN THE PROFESSION.

BY J. F. SIDDALL.

It is not to boast of my long experience, and consequently wonderful skill, that I present a few thoughts that have suggested themselves to me in taking a retrospect of my experience in dentistry; for ten years is too short a time to do more than *start* a man well in our profession, without giving him much to *ventilate.*

I think there is no greater humbug than that of parading our long experience before the public as sufficient evidence of skill; not that it deceives the people; for they know full well that it takes something more than *age* to make a man in any business.

The professional man whose great recommendation is his long experience, may be greatly comforted as he grows older, and feel he is becoming wiser, but others may *not see it.*

This weakness is by no means confined to the older members of the profession; for it takes some of us but a very short time to have had a very great experience. Young men are proverbially distinguished for their wisdom, however, and young dentists sometimes—*may be.*

However it may be with us as dentists, it certainly is true of us as a profession that the years of experience, and especially the last *ten* years, have been rapidly developing skill and efficiency heretofore unknown.

Ten years ago we had no organizations, but were one chaotic mass, every fellow for himself. Each man had his little chest under lock and key, which was supposed to contain a great deal of something, no one knew just what, nor whom it was designed to bless, unless it should be the man who *kept it.*

The last ten years have been breaking open these private treasuries for the benefit of the profession at large; and the true dentist has thrown away his key, and hung out his latch-string, and inscribed over it, "walk in;" and we find this new arrangement has a two-fold advantage, viz: we impart our good methods and improvements to others and benefit them; and in trying to impart our bad ones, we find out that they are bad, and we are benefitted.

Thus, from the multitude of experiences and practices, sifted and refined, we come to have *recognized modes of practice.* Our social intercourse we thus have is of great advantage. It stimulates us to more earnestness and energy in our work, and makes us feel that we are not alone, that our profession is an honorable and useful one, and that there are honorable and useful men in it; and if we would be worthy of a place in its ranks, we must be active.

No *live* man will be satisfied, after meeting a few times in convention and hearing the discussions, and witnessing our clinics, with plodding along out of the ranks and in the rear; but if possible, will be up with the times, and will do what others are doing, and do it as well.

Our improved methods of treating, filling, and *saving* the teeth we once extracted, and of more thoroughly performing all other operations, (which we most certainly are doing) in connection with the state of our currency, make it necessary to charge for our services what sometimes seems to be extortionary prices. To avoid this censure and meet the public demand, though oftener, because it "pays" many do cheap work.

This may be justifiable in some cases, but as a general rule is to be condemned. Emerson says, "nothing for nothing;" and so it is the world over, that which costs but little is generally of little value.

To be sure, we may undervalue those low-priced operations, and the man who makes them; but, making all allowance for prejudice, who of us ever knew a first-class operator to work for the lowest prices?

That dentists receive too large a remuneration, if it be true of any class, it certainly is of those who do the poorest work, and especially in the operative department.

I think if any professional man is well compensated, the man who saves teeth should be. It takes much time and close application to acquire the necessary skill; and with this acquired, there are so many things to be considered, and difficulties to be met and overcome in filling teeth, that unless you have the physical ability to *work*, and keep your mind on your work, and control yourself and your patient, you cannot successfully opperate. And then, *no* man, even in full health, should work more than six hours per day at the chair. If he goes much beyond this, he will suffer for it. And as we grow older, or have impaired health, we must be content with doing less. When our muscles begin to fail, our eyes to grow dim, and the right hand to forget its cunning, we can *do* but little, however fully we may possess our mental vigor.

If we were in almost any other occupation, we could endure longer. The farmer, the manufacturer, the merchant, can employ help, and vigorously pursue his calling to a much greater age. Now, I think it but just for the dentist, in establishing his prices, to consider all these matters. And yet there are other interests to be thought of. He must consider charitably the ability of his patients—must strictly apply the golden rule in every case. Before he charges a poor sewing girl, or a mechanic with a large family to support, for one day's work as much as either of them could save in a month, he must inquire: is this just? Is this doing as we would be done by?

Let us consider first, that though our Creator has made us equal in some respects, in others we differ. To some he has given one talent, to others ten; and we are accordingly responsible. In nothing, perhaps, do we differ more than in our ability to accumulate property. One will make his time worth $50 per day, another, equally as intelligent, and as highly respected, may exert himself as much, be just as honest and honorable, *and no more so*, and may save 50 cts. per day. To be sure, men may gain wealth by unfair and dishonorable means, and so may they poverty; for there is probably as much dishonesty among the poor as among the rich, and both alike are culpable.

The man who digs ditches at $2 per day, is just as much bound to be faithful, as he who employs him is, who may make $20 per day out of his contract.

This difference in remuneration, which different occupations bring, everywhere exists, and I think rightfully and wisely.

When the young man steps forth to take his place in the world, he chooses his occupation according to his taste, or what he dare undertake, and succeeds or fails, as he has, or has not the mettle to carry him through. Positions that "pay well" are generally obtained at a risk. "Nothing ventured, nothing won."

To become a good dentist, requires some venture; a man may fail; and if he succeeds, it is not through luck, a rich uncle, or any one's influence; but by his own persistent perseverance. If he gains wealth, it is seldom, if ever, by his profession; and yet he may, with a good practice, gain a competency, and live as well as his neighbors.

Now, in regard to the sewing girl, the mechanic and the class of patients they represent. I think it may often be our duty to work for such as they at a discount; but our own convictions must decide *when* and to *whom* such favors must be bestowed. The merchant has no more reason to expect me to take the case of his hired girl, or poor sister into hand and do work worth $10 or $15, which would cost me one day's time, for what one of them could earn in a day, than I have to expect him to bestow upon my poor friend a piece of goods that may represent one day's time of his, for a like remuneration.

As a general rule, no one has reason to expect that for which they cannot pay the customary price. The poor can get along without teeth much better than without many other things no one expects to see bestowed upon them gratis.

And yet, "the poor ye always have with you, and when ye will ye may do them good." I would, by no means say, aught against any man giving to the poor: for "he that giveth to the poor lendeth to the Lord." Our best friends, and most appreciative, are sometimes poor; and I would advocate a liberal fee-bill, *partly* on their account, that we may thus be *able* to favor them when we choose.

I suppose, to a considerable extent, *custom* determines the fees we should charge; and yet, we have the power and responsibility, to some extent, of fixing our own prices on each operation; and, when we are so disposed, we may be unjust.

No class of men have a better chance to swindle than we; and still we have the golden opportunity of rendering many fold, in real blessing for what we receive.

Since we have become organized throughout the country, we are determining pretty much wholly for ourselves what our customary fees shall be. This, in many respects, is a good thing; for who can judge better than the man who renders a service, what he can afford it for? But it is well for us to remember that we are not sanctified yet; and self interest *may* lead us astray.

Before our organizations existed, we were, to a great degree, at war with each other on prices, as well as concerning other matters; sometimes (I am sorry to say it) working for less than we should, for the sake of taking business away from each other. Now, this evil, as we are becoming acquainted with, and interested in each other, is disappearing; and I have thought that at present there might be danger in the other direction. If our patients, and the public in general, get the impression that we are associating together, to work for our own interests, regardless of theirs, and demand unreasonable fees for our services, it will surely work against us, and only react in favor of those who hold aloof from our organizations, and in favor of quackery in general; and against the passage and enforcement of any law which otherwise would be for the general good.

Therefore, let us take such a course as will keep the *people* in sympathy with our *profession*, rather than with the quacks.

Let us *do* nothing, *say* nothing, *attempt* nothing, in our intercourse with each other, that we would not have our patients fully understand. Let us remember *their* interests as well as our own.

Let us strive to make our operations of the first order; and charge a *reasonable*, and yet an *ample* fee for our services. Always avoiding great extremes in either direction; ever discharging faithfully our obligations to each other, and our profession, ourselves and our patrons.

